# Paraneoplastic limbic encephalitis associated with mixed olfactory neuroblastoma and craniopharyngioma: A case report and literature review: Erratum

**DOI:** 10.1097/MD.0000000000011575

**Published:** 2018-07-13

In the article, “Paraneoplastic limbic encephalitis associated with mixed olfactory neuroblastoma and craniopharyngioma: A case report and literature review”,^[1]^ which appeared in Volume 97, Issue 24 of *Medicine*, Dr. Dai Sato's degree was left off and should be MD.
